# Pimavanserin: A Novel Antipsychotic With Potentials to Address an Unmet Need of Older Adults With Dementia-Related Psychosis

**DOI:** 10.3389/fphar.2020.00087

**Published:** 2020-02-26

**Authors:** Ismaeel Yunusa, Marie Line El Helou, Saud Alsahali

**Affiliations:** ^1^ School of Pharmacy, Massachusetts College of Pharmacy and Health Sciences, Boston, MA, United States; ^2^ School of Pharmacy, Lebanese American University, Byblos, Lebanon; ^3^ Unaiz College of Pharmacy, Qassim University, Qassim, Saudi Arabia

**Keywords:** pimavanserin, dementia, behavioral and psychological symptoms of dementia, dementia-related psychosis, neuropsychiatric symptoms of dementia, Alzheimer's disease, Antipsychotic, Atypical antipsychotic

## Abstract

Dementia affects more than 40 million people worldwide. When it is accompanied by psychosis, symptom management is especially challenging. Although no drug has been approved by the US Food and Drug Administration (FDA) for psychosis in patients with dementia, atypical antipsychotics are used off-label in severe cases in patients who do not respond to non-pharmacological interventions. However, antipsychotic use in elderly patients with dementia-related psychosis (DRP) is associated with adverse reactions including motor function disorders, cognitive impairment, cerebrovascular events, and increased risk of death. In 2017, the US FDA granted breakthrough therapy designation to the new antipsychotic pimavanserin for the treatment of DRP. Topline result of the pivotal phase III HARMONY (NCT03325556) trial suggests that pimavanserin reduces the relapse of psychosis by 2.8-folds compared to placebo. This favorable result may open path for the potential approval of pimavanserin in DRP. In this review, we discuss the pharmacological activity, clinical efficacy and safety of pimavanserin as a novel atypical antipsychotic with potentials to address the unmet needs of older adults with DRP.

## Background

It is estimated that 44 million people worldwide are currently living with dementia and this number is expected to triple by 2050 (Alzheimers.net, 2013). In addition to cognitive impairment, dementia is often accompanied by behavioral and psychological symptoms. Indeed, up to 90% of people with dementia experience behavioral and psychotic symptoms of dementia (BPSD) throughout the course of the disease (Selbæk et al., 2013). These symptoms include depression, psychosis, aggression, and agitation and they can lead to complications that further reduce the patient’s quality of life. Different subtypes of dementia diseases including Alzheimer’s disease (AD), dementia with Lewy bodies, frontotemporal dementia, and vascular dementia exhibit dementia-related psychosis (DRP). These psychotic symptoms present as delusions, with a reported prevalence ranging between 30%–40% and hallucinations, with a prevalence of 5%–20% (Flint, 1991; Sultzer, 2004). According to Reeves and colleagues (2012), DRP is a “logical attempt to understand the environment” in the context of a degraded cognitive integrity. Psychosis aggravates the clinical course of dementia and creates profound stress on caregivers and family members, which may lead to placing patients in long-term care facilities earlier in the course of the illness (Koppel and Greenwald, 2014).

To date, no drug has received approval by the US Food and Drug Administration (FDA) for treating DRP. Given the severity and high prevalence of BPSD and the lack of FDA-approved pharmacological treatment, many classes of drugs (antipsychotics, antidepressants, and anticonvulsants) have been utilized off-label for the management of BPSD. The most widely used and most effective drugs for this purpose are the atypical antipsychotics, including aripiprazole, risperidone, olanzapine, and quetiapine (Alzheimer’s Association, 2013.). However, antipsychotic use in elderly patients with DRP is associated with adverse reactions that include motor function disorder, cognitive impairment, cerebrovascular events (stroke and transient ischemic attack), and increased risk of death (Schneider et al., 2006; Huybrechts et al., 2012; Zhai et al., 2016). In this review, we focus on pimavanserin, a novel antipsychotic that was granted the FDA’s breakthrough therapy designation for the treatment of DRP in 2017 (Tan, 2019). This designation indicates the FDA will fast-track its review and development.

## Pharmacology of Pimavanserin

Pimavanserin is the active pharmaceutical ingredient of Nuplazid^®^, which was approved by the FDA in 2016 for the treatment of hallucinations and delusions associated with psychosis in patients suffering from Parkinson’s disease. Its exact mechanism of action is unclear. The drug exhibits a combination of inverse agonist and antagonist activity at the serotonin 2A receptors (5-HT2A) and, to a lesser extent, at the 5-HT2C receptors in the central nervous system, which is believed to contribute to its antipsychotic activity. Pimavanserin is absorbed in the gastrointestinal tract and is highly bound to plasma protein (approximately 95%). Following the administration of a single 34-mg dose of pimavanserin, the time to maximum plasma concentration is six hours, the half-life is approximately 57 h and the mean (standard deviation, SD) apparent volume of distribution is 2,173 (307) L. Pimavanserin is metabolized in the liver predominantly by cytochrome P450 (CYP3A4 and CYP3A5) and does not cause significant CYP3A4 inhibition or induction. It has a major active N-desmethylated metabolite AC-279, whose half-life is 200 h (Paspe Cruz, 2017).

Pimavanserin is the first atypical antipsychotic that does not induce clinically significant antagonism of adrenergic, dopaminergic, histaminergic, or muscarinic receptors (see **Table 1** for comparison with other atypical antipsychotics) (Paspe Cruz, 2017). This may explain the absence of movement-related disorders seen with other antipsychotics. The most frequent adverse reactions are peripheral edema (7%), nausea (7%), and state of confusion (6%) (FDA, 2018).

**Table 1:** Receptor selectivity of pimavanserin and other atypical antipsychotics (Nasrallah, 2008; Hacksell et al., 2014; Mauri et al., 2014; Kusumi et al., 2015; Siafis et al., 2018).

Atypical Antipsychotic*	Receptors																	
	5-HT_1A_	5-HT_2A_	5-HT_2B_	5-HT_2C_	α1A	α1D	α2A	α2B	α2C	D1	D2	D3	H1	M1	M2	M3	M4	M5
Amisulpride	✓	✓		✓	✓	✓	✓	✓		✓	✓	✓	✓	✓	✓	✓	✓	✓
Aripiprazole	✓	✓		✓	✓	✓	✓	✓	✓	✓	✓	✓	✓	✓	✓	✓	✓	
Asenapine	✓			✓	✓	✓				✓	✓	✓	✓					
Brexpiprazole	✓	✓	✓	✓	✓		✓	✓	✓	✓	✓	✓	✓					
Clozapine	✓	✓	✓	✓	✓	✓	✓	✓	✓		✓		✓	✓	✓	✓	✓	✓
Iloperidone	✓	✓		✓	✓	✓					✓	✓	✓					
Lurasidone	✓	✓		✓	✓	✓												
Olanzapine		✓	✓	✓			✓	✓	✓	✓	✓	✓	✓	✓			✓	✓
Quetiapine		✓	✓		✓				✓		✓	✓	✓	✓			✓	✓
Paliperidone	✓	✓		✓		✓	✓				✓	✓		✓				
Pimavanserin		✓		✓														
Risperidone	✓	✓	✓	✓	✓	✓	✓	✓	✓	✓	✓	✓	✓	✓	✓	✓	✓	✓
Ziprasidone	✓	✓		✓		✓	✓	✓	✓	✓	✓	✓	✓	✓	✓	✓	✓	✓

D, dopamine; M, muscarinic; 5-HT, serotonin; α, alpha adrenergic receptor.

*The symbol ✓ indicates that the antipsychotic has an affinity for the receptor in the corresponding column.

## Approval for Treating Parkinson’s Disease-Related Psychosis

The efficacy of pimavanserin for treating hallucinations and delusions associated with Parkinson’s disease was demonstrated in a 6-week, randomized, placebo-controlled, parallel-group phase III study by Cummings et al. The study randomized 199 patients (mean age 72 years) with Parkinson’s disease psychosis (hallucinations and/or delusions) to receive either pimavanserin 34 mg daily or placebo. The outcome was assessed with the Parkinson’s disease-adapted scale for assessment of positive symptoms (SAPS-PD). The group receiving pimavanserin showed a significantly improved SAPS-PD score at week 6 compared to the group receiving placebo. Indeed, a 5.79 point improvement [least square (LS) mean change] in the SAPS-PD score was observed with pimavanserin compared to a 2.73-point improvement for placebo (treatment difference of 3.06 points; p = 0.001). Overall, pimavanserin was well tolerated with no significant safety concerns or worsening of motor function (Cummings et al., 2014).

A post-hoc subgroup analysis of the results revealed a significant improvement in the SAPS-PD score of PD patients with cognitive impairment (n = 50) treated with pimavanserin compared to those on placebo, with no adverse effect on cognition. Moreover, pimavanserin led to better improvement in the SAPS-PD score in the subgroup of patients with cognitive impairment (treatment difference of 5.71 points; p = 0.002) than in the overall PD population (treatment difference of 3.06 points; p = 0.001), suggesting a more robust effect in the former group than the latter (Cummings et al., 2018). Based on this finding, it was extrapolated that pimavanserin may also be a potential treatment for dementia with Lewy bodies, which is also characterized by movement disorders and is associated with psychotic symptoms in 75% of patients (Lyketsos et al., 2002). Despite data from this subgroup analysis suggesting a better safety profile, pimavanserin, like other antipsychotics, carries a black box warning stating that “elderly patients with DRP treated with antipsychotic drugs are at an increased risk of death” (FDA, 2018).

## Clinical Evidence From Trials In Dementia-Related Psychosis

A phase II randomized, double-blind, placebo-controlled, single-center clinical trial assessed the safety and efficacy of pimavanserin 34 mg daily versus placebo for the treatment of AD psychosis. Completed in 2016, the study included 181 participants (mean age 86 years) from multiple affiliated nursing-home sites across the United Kingdom (UK). The primary endpoint was the mean change in the Neuropsychiatric Inventory-Nursing Home version (NPI-NH) psychosis score, from baseline to week 6. At week 6, patients in the pimavanserin group showed significant improvement in the NPI-NH psychosis score compared to patients in the placebo group. Mean change in the NPI-NH psychosis score from baseline at week 6 was −3.76 points [standard error (SE), 0.65] for pimavanserin and −1.93 points (SE, 0.63) for placebo [mean difference −1.84 (95% CI –3.64 to −0.04); p = 0.045] (Ballard et al., 2018). However, in this trial, a significant difference in efficacy between pimavanserin and placebo was not seen at 12 weeks of treatment (**Table 2**). Although agitation was higher in patients receiving pimavanserin (21%) than in those receiving placebo (14%), the overall adverse event profile was similar in the two groups. No detrimental effect was observed on cognition or motor function in either group (Ballard et al., 2018).

**Table 2:** Summary of completed and ongoing clinical trials for pimavanserin in dementia-related psychosis.

Trial	Treatment Comparison		Trial Phase	Blinding	Sample size	Symptom	Outcome	Relative Treatment Effect (95% CI)	
	Intervention	Comparator						Effect size (95% CI)	P-value
SERENE (ClinicalTrials.gov identifier:NCT02992132)	Pimavanserin 20 mg (PO, qd) and 34mg (PO, qd)	Placebo (PO, qd)	2	Double-blind	111	Aggression and Agitation	**Primary:** Cohen-Mansfield Agitation Inventory (CMAI) **Secondary:** Zarit Burden Interview	N/A	N/A
HARMONY (ClinicalTrials.gov identifier:NCT03325556)	Pimavanserin 20 mg (PO, qd) and 34mg (PO, qd)	Placebo (PO, qd)	3	Double-blind	392	Psychosis	**Primary:** Time from randomization to relapse in the double-blind period **Secondary:** Time from randomization to discontinuation from the double-blind period for any reason	Primary endpoint HR, 0.353 Secondary endpoint HR, 0.452	Primary endpoint, 0.0023* Secondary endpoint, 0.0024*
ClinicalTrials.gov identifier:NCT03118947	Pimavanserin 20 mg (PO, qd) and 34mg (PO, qd)	N/A	2	Open label	79	Aggression and Agitation	**Primary:** Treatment emergent adverse events (TEAEs)	N/A	N/A
ClinicalTrials.gov identifier:NCT02035553	Pimavanserin tartrate 40 mg (PO, qd)^a^	Placebo (PO, qd)	2	Double-blind	181	Psychosis	**Primary:** Neuropsychiatric Inventory-Nursing Home Version (NPI-NH)	At 6 weeks: MD, −1·84 (−3·64, −0·04) At 12 weeks: MD, −0·51(−2·23, 1·21)	0·045 (at 6 weeks) 0·561 (at 12 weeks)

CI, confidence interval; HR, hazard ratio; MD, mean difference; N/A, not available or not applicable; PO, taken orally; qd, daily.

^a^Pimavanserin tartrate 40 mg is equivalent to 34 mg free base pimavanserin.

*one-sided p value.

Two other trials are currently evaluating pimavanserin in patients with dementia: the SERENE trial (ClinicalTrials.gov number: NCT02992132) and the HARMONY trial (ClinicalTrials.gov number: NCT03325556). The SERENE trial is a double-blind, placebo-controlled phase II trial examining the safety and efficacy of pimavanserin to treat agitation and aggression in people with AD. Patients are given either 34 or 20 mg of daily pimavanserin, or a placebo for three months. Patients who successfully complete the SERENE trial are eligible to take part in an open-label extension study (ClinicalTrials.gov number: NCT03118947) that assesses the safety and tolerability of 34 or 20 mg of pimavanserin taken for a year. The SERENE trial was completed in February 2018, and its extension was completed in February 2019 (ClinicalTrials.gov, 2019a; ClinicalTrials.gov, 2019b; Tan, 2019). To our knowledge, no data from this phase II clinical trial and its extension have been published to date.

The HARMONY trial is a phase III, double-blind, placebo-controlled study evaluating the efficacy of pimavanserin versus placebo in preventing a relapse of psychotic symptoms in patients with DRP who are stabilized after 12 weeks of open label pimavanserin treatment. Unlike the phase II trials that were restricted to patients with AD, this phase III trial also includes patients with Lewy body dementia, Parkinson’s disease dementia, frontotemporal dementia, and vascular dementia. During an initial open-label, 3-month period, all patients receive 34 mg of pimavanserin daily. After this period, patients are randomized to continue receiving 34 mg of pimavanserin daily, to switch to 20 mg daily, or to be given a placebo. HARMONY started in September 2017, and recent topline results suggested that pimavanserin reduced the risk of relapse of psychosis by 2.8-fold compared to placebo in patients with DRP (see **Table 2**) (Acadia Pharmaceuticals, 2019; Tan, 2019).

### Potential Off-Label Use

An International Delphi consensus panel of 11 experts in the management of BPSD chose risperidone as the recommended pharmacological option for this indication, and regarding future treatments, the greatest priority was placed on pimavanserin (Kales et al., 2019). Given its approval for Parkinson’s-related psychosis, its selection by the consensus panel for future treatments, and the promising published results from the phase II and III clinical trials indicating pimavanserin’s favorable efficacy/safety profile over placebo than the current pharmacological options, pimavanserin is likely to be used off-label for the treatment of DRP pending its potential approval. However, to our knowledge, there is no published data on the status of its likely off-label use in clinical practice.

## Conclusion

Preliminary clinical evidence suggest that pimavanserin may have a positive benefit-risk profile for the short-term treatment of DRP, which contributed to it’s designation by the FDA as a breakthrough therapy for this indication. Results from the phase III HARMONY trial will open the path for the FDA’s priority review and a potential approval of pimavanserin for DRP.

## Author Contributions

IY, ME, and SA determined the outline, reviewed the literature, wrote, verified, and approved the manuscript.

## Conflict of Interest

The authors declare that the research was conducted in the absence of any commercial or financial relationships that could be construed as a potential conflict of interest.
